# Communicating psychosocial well-being in motor neurone disease to staff: results from a World Café approach

**DOI:** 10.1007/s11136-019-02193-x

**Published:** 2019-04-30

**Authors:** Clarissa Giebel, Gillian Medley, Sandra Smith, Maria Thornton, Moira Furlong, Michelle Ennis, Carolyn Young

**Affiliations:** 1 Institute of Population Health Sciences, University of Liverpool, Waterhouse Building B Block, Brownlow Street, Liverpool, L69 3GL UK; 2 NIHR Collaboration for Leadership in Applied Health Research and Care North West Coast, Liverpool, UK; 3 The Walton Centre NHS Foundation Trust, Liverpool, UK; 4 Motor Neurone Disease Association, Northampton, UK; 5 Faculty of Health and Medicine, Lancaster University, Lancaster, UK

**Keywords:** Motor neurone disease, Staff training, Quality of life, Psychosocial well-being, Service evaluation, Health inequalities

## Abstract

**Objective:**

Little to no research has evaluated staff training and its effects on the well-being of people with MND. The aim of this study was to assess how educating multi-disciplinary staff about psychosocial well-being in MND can change approaches to working with people with MND.

**Methods:**

Multi-disciplinary staff attended a half-day workshop to receive training on psychosocial well-being in people with MND and to discuss QoL issues using the World Café approach. Prior to the workshop and 2 weeks post-workshop, staff completed a questionnaire on their knowledge of this topic. A selection of staff completed a follow-up interview 2 months later to assess changes in their practice.

**Results:**

19 staff, including dieticians and occupational therapists, attended the workshop and completed the pre-workshop questionnaire. Ten filled in the post-workshop questionnaire and were interviewed. Clinicians identified six strategies/barriers of improving communication amongst MND staff, suggesting the need for better collaborative working, raising awareness of psychological and emotional issues in MND and barriers to service access due to health inequalities, amongst others.

**Conclusions:**

This workshop raised staff awareness on communicating QoL in MND. Future work needs to look into implementing this training in clinical practice and evaluate their impact on QoL in MND.

## Introduction

In the UK, 5000 people are diagnosed with motor neurone disease (MND) [1]. MND is characterised by muscle weakness, fatigue and speech problems, and people suffer from swallowing and respiratory impairment. To provide adequate care for people with MND, a multi-disciplinary team (MDT) addresses physical, emotional and social needs of people with MND [2, 3]. Whilst physical symptoms are prominent, quality of life (QoL) is receiving a great deal of attention [4, 5]. Indeed, symptom management and addressing QoL through a MDT approach are suggested to be the best treatment practices [6].

Receiving a diagnosis of MND can come as a shock to patients and family members, and people with MND experience a range of emotions when being communicated the diagnosis [7]. Addressing the emotional needs of people with MND influences their quality of life (QoL) [8]. Research has shown that physical symptoms are less important than psychosocial and emotional needs when addressing QoL in MND [9, 10], yet there is still limited research [10]. For example, functioning and social support were found to predict health-related QoL in MND [11], whilst disease duration and socioeconomic status were not. Therefore, more attention needs to be paid to the emotional and psychosocial needs in MND.

Different types of interventions have been investigated, including expressive disclosure, cognitive behavioural therapy and hypnosis [12–14]. However, there is no conclusive evidence as to which interventions are effective in improving the well-being by addressing psychosocial aspects [10, 15]. One avenue to pursue would be educating staff. Whilst not investigated in MND, this is a frequently explored avenue in dementia care [16, 17].

The first objective of this study was to educate clinical staff about psychological and social well-being in MND to establish whether education may change their clinical practice and increase their focus on psychosocial issues when managing QoL. The second objective was to gather insights from multi-disciplinary staff on how communication could be improved amongst staff teams. Considering the sparsity of interventions on staff education regarding psychosocial issues in MND, this service evaluation appears to be one of the first to evaluate the effects of addressing these needs through staff.

## Methods

### Participants and recruitment

The Health Research Authority indicated that the project was not a research project but a staff service evaluation, and therefore did not require ethics (IRAS 243013). The Research and Development department at the Walton Centre NHS Foundation Trust approved the study. A mix of clinical healthcare professionals working with people with MND were recruited via a snowball technique. Staff from community health and social services across the Liverpool region were contacted via e-mail.

### Data collection

Staff completed a pre-workshop questionnaire about their discipline, length of service, areas of interest in MND, receipt of MND training in the past 2 years and knowledge on factors influencing QoL in MND. At the beginning of the workshop, participants provided written informed consent before completing the pre-workshop questionnaire and participating in the workshop.

By using a World Café approach [18], talks were provided on QoL issues in MND and results relating to the TONiC study relating to MND [19], and about raising awareness of health inequalities. Attendees were divided into four groups of three to five people, moving between tables every 20 min. Facilitators at each table ensured the write-up of notes resulting from each group discussion.

Two weeks post workshop, staff completed a follow-up questionnaire to compare their knowledge about QoL in MND. 2 months post-attendance staff who had expressed an interest were interviewed about their learning at the workshop and views on QoL in relation to their practice in MND. Telephone interviews lasted approximately 10 min and were audio-recorded and transcribed.

### Data analysis

Data from 19 pre- and 10 post-workshop questionnaires were analysed using frequency analysis. Qualitative information from the 10 interviews was analysed using thematic analysis [20]. A public adviser helped to further inform the coding and theme development.

### Public involvement

Two former MND carers were involved in the concept, design, analysis and dissemination. Both attended regular team meetings and workshops, hosted by the NIHR CLAHRC NWC. Advisers were reimbursed according to NIHR INVOLVE guidelines [21].

## Results

Nineteen clinical staff working with people with MND attended the workshop, including dieticians, physiotherapists, psychologists, occupational therapists, speech and language therapists and assistive technology representatives. Attendees had on average 12.5 years (± 9) [0.1–29 years] of experience of working with people with MND.

### Pre- and post-workshop questionnaire

The post-workshop questionnaire results demonstrated improved knowledge of QoL in 9 out of 10 people. Respondents detailed the workshop had provided opportunity to network and share experiences of managing MND and increased the understanding of QoL and health inequalities. Staff highlighted that improved communication may address QoL, and that QoL is different for each individual and therefore no assumptions should be made by professionals. Staff also raised the financial implications that caring for MND brings.

### World Café workshop and interviews

Staff overall engaged in more communication about QoL within their teams after the workshop. Six themes with several sub-themes emerged from the workshop notes and interviews (Table 1), with quotes provided in Table 2.

**Table 1:** Identified themes and sub-themes from World Café workshop and post-workshop interviews

Main themes	Sub-themes
Collaborative and creative working	Team enhancement MDT Rainbow caring MDT Rainbow support Service flexibility Developing new horizons
Improved specialist and referral links	Journey to experts Pathway needs psychology Enhance referral process Quality referral Right time right place referral Self-referral
Knowledge base/experience	Learning barriers Enhancing specialised MND knowledge Sharing knowledge Sharing pathway knowledge The learning trajectory Confidence to manage MND
Promote information and understanding throughout trajectory	Enlightened informing Better working Condition adjustment Service flexibility Right time right place
Promote individuality of PwMND and family	Keeping on track Right approach for PwMND Making more of what we’ve got Barriers to living with MND
Pathway predicaments	Right time right health professionals Pathway inertia Chronological Care Postcode lottery Pathway building

**Table 2:** Quotes by identified theme

Theme	Quotes
(1) Collaborative and creative working (2) Improved specialist and referral links	“I often think that one of the more difficult things patients manage really struggle from is a psychological point of view in terms of coming to terms with their diagnosis” “when you are trying to get people to acknowledge their functional difficulties that they are having, if they have not come to terms with their diagnosis, it is very difficult to get them to forward plan and then you end up crisis managing”
(3) Knowledge base/experience	“at the moment there is not really a forum for CPD within the multi-disciplinary teams, we have regular MDT meetings to specifically discuss patients and then within each individual team have their own CPD sessions, but may be it would be good to have multi-disciplinary CPD sessions, or like a case review, as part of the CPD, to support what worked and what didn’t”
(4) Promote information and understanding throughout trajectory	“because we are all so spread out and we are dealing with community, social services and the acute services and trying to coordinate this sort of care package for these patients and we are probably all doing it in isolation, but not talking to each other and it is finding a method of how that would work and it sort of happens anyway”
(5) Promote individuality of people with MND and family (6) Pathway predicaments	“Also thinking about locations, we have had a patient before who can be on the border and I think I have made an effort to actually make sure I would see her as often as I would see someone living closer within an area for me to get to, because obviously if she is referred to our service it should be the same regardless of where.” “a shame we cannot […] have a pot of money that we can address the needs of motor neurone disease sufferers quickly. That is the only thing, we are always playing catch up I think with the illness and it is a shame that they cannot have like an individual pot of money so they get what they need at the right time”

*Improved collaboration* and *establishing specialist and referral links* with partners emerged as some of the primary strategies, including the need to provide specialist services in the home environment, and referring to appropriate post-diagnostic services. Participants specifically raised the importance of psychological support. If people with MND struggle accepting their condition, it can be difficult for them to access appropriate support. Often psychologists are missing from multi-disciplinary teams, so that the emotional needs of people with MND can lack support.

Staff also highlighted the need for *improving knowledge and raising awareness,* as some staff are not aware of specific post-diagnostic support services and treatments. Moreover, general practitioners were flagged up for not being always aware of the best ways of supporting the health and social care needs in MND, suggesting the need for enhancing specialised MND knowledge. This lack of knowledge was also highlighted for care home staff, who are said to often only have limited knowledge about MND. Overall, staff raised physical issues and supporting people with MND and their families to a greater extent than psychosocial issues in MND.

The need for *appropriate information sources and promoting the understanding of MND throughout the trajectory*, not only at diagnosis, was highlighted. It was detailed that this is not solely the responsibility of professionals. Instead, information provision needs to be considered in novel ways by making more of what is already available within communities and joining up with health and social care agencies.

To *promote individuality* and overcome *pathway predicaments,* people with MND and their carers need to be supported depending on their individual needs. When discussing access to care and support services, staff highlighted the fact that people receive different levels of care depending on their socioeconomic status and the neighbourhoods they live in. Depending on whether patients live in an advantaged or disadvantaged area, they receive different support from MDTs. This can result in people reaching crisis point first in disadvantaged areas before accessing services.

Experiencing health inequalities are also said to be reflected in accessing specialised equipment due to a lack of funding, either personally or from the councils. Staff referred to the loss of household income as a result of receiving a MND diagnosis, and the implications this can have for the person with MND and their family. This causes many issues in managing MND effectively, with needs changing quickly. Occupational therapists reiterated this need for equipment, and the frustration it can cause with their day-to-day work.

## Discussion

This World Café workshop successfully raised knowledge about QoL in MND in staff, and encouraged discussions of QoL in staff teams, thereby addressing our main objectives. This is a service evaluation, so that findings are not nationally representative. However, this is one of the first reported methods of addressing QoL, with staff suggesting several strategies of improving team communication on QoL.

One area of clinical practice that was considered to be lacking was emotional and psychological support by psychologists. To better deal with the range of emotions and psychological responses experienced after a diagnosis, a more person-centred approach from the diagnosis onwards is recommended [22]. Therefore, it is important for MDTs to provide different models of psychological support, guided by a psychologist.

Health inequalities in accessing MND care was also raised. Considering the large costs associated with MND, approximately £10,000 per year [23], this may not be surprising, although no previous research has addressed this topic. According to staff, health inequalities are often reflected in differences in accessing new equipment or paid care. Due to the quick deterioration of functioning in MND, people need various types of equipment to support them in their daily lives, such as wheelchairs or handrails. With equipment often being costly [24], people from disadvantaged backgrounds are less able to purchase these and likely to depend on their local council. However, some councils have more money available to fund MND care than others, so that people almost experience a ‘postcode lottery’ depending on their neighbourhood.

Other ways to improve QoL in MND were suggested to be promoting understanding throughout the MND trajectory. People with MND have previously reported to often lack information about their symptoms at the time of diagnosis [25]. This would require the MDT to be in constant contact with the person. In particular, information on psychosocial and cognitive symptoms should best be provided by a psychologist, which supports the need for a psychologist as part of the team further.

This project solely focused on staff perspectives, and did not measure the QoL of people with MND. Therefore, the only factor of change is qualitative and subjective information provided by staff. Future evaluations will have to assess QoL pre- and post-workshop to establish whether staff education has changed well-being in MND.

## Conclusions

This is one of the first reported methods to address psychological and emotional needs in MND via improving staff communication. The next step will be to implement findings in practice, by producing an implementation plan, to be able to replicate this service in other clinical settings to also help improve QoL for people from any socioeconomic background.
